# Influenza-specific antibody-mediated and complement-dependent cellular cytotoxicity-inducing antibodies in vaccinated and infected pigs

**DOI:** 10.3389/fimmu.2025.1600761

**Published:** 2025-06-30

**Authors:** Mithilesh Singh, Gabriela Mansano do Nascimento, Sankar Renu, Dina Bugybayeva, Olaitan C. Shekoni, Raksha Suresh, Jennifer Schrock, Sara Dolatyabi, Diego G. Diel, Prosper N. Boyaka, Gourapura J. Renukaradhya

**Affiliations:** ^1^ Center for Food Animal Health, Department of Animal Sciences, College of Food, Agricultural, and Environmental Sciences, The Ohio State University, Wooster, OH, United States; ^2^ Department of Population Medicine and Diagnostic Sciences, Animal Health Diagnostic Center, College of Veterinary Medicine, Cornell University, Ithaca, NY, United States; ^3^ Department of Veterinary Biosciences, College of Veterinary Medicine, The Ohio State University, Columbus, OH, United States

**Keywords:** swine influenza a virus, intranasal vaccine, chitosan nanoparticle, antibody functions, ADCC and CDC

## Abstract

In addition to neutralizing activity, antibodies can contribute to protection against viral infections through antibody-dependent cellular cytotoxicity (ADCC) and antibody-mediated complement-dependent cell cytotoxicity (CDC) mediated via Fcy receptors. Swine is a suitable large-animal biomedical model for influenza research, because it is a natural host for influenza like humans exhibiting comparable clinical and immunological responses. Unfortunately, there are currently limited insights into ADCC and CDC functions to swine influenza A virus (SwIAV) in pigs due to lack of adequate immunological tools. Therefore, the present study was aimed at optimizing the ADCC and CDC assays to evaluate the cytotoxicity mediated by virus-specific antibodies in response to vaccination of pigs with chitosan nanoparticle-based inactivated monovalent and commercial multivalent SwIAV vaccines administered through intranasal and intramuscular route, respectively. Using these assays, we quantified and compared the antibody-mediated cytotoxicity induced in pigs by intranasal chitosan nanoparticle-based inactivated monovalent whole SwIAV vaccine and intramuscular administered commercial multivalent SwIAV vaccine. Our results revealed that maternal antibody-positive pigs following vaccination with whole inactivated virus failed to elicit specific ADCC-mediating antibodies, but production of CDC antibodies was not affected. However, after exposure of vaccinated animals to challenge infection, high levels of ADCC antibodies were elicited. Further, it was observed that the function of virus-specific neutralizing and non-neutralizing antibodies are influenced by route of vaccination (intranasal versus intramuscular), vaccine type (monovalent versus multivalent) and adjuvant formulation. Overall, we observed a positive trend among the magnitude of ADCC, CDC, antibody avidity, Nabs, and HA inhibition (HAI) antibody responses in vaccinated and influenza virus-infected pigs. In conclusion, measuring ADCC- and CDC-mediating antibodies in pigs is important for evaluating the protective immunity against influenza by vaccines. Monitoring the function of both virus-neutralizing and non-neutralizing antibodies in vaccinated animals aid in the development of innovative cross-protective vaccine formulations to fight against constantly evolving influenza viruses.

## Introduction

Swine influenza A virus (SwIAV) causes an acute respiratory illness in pigs, with periodical zoonotic infections in humans from few SwIAV strains (1), like the 2009 pandemic influenza (2). Similar to humans, the most common circulating subtypes of IAV in swine population are H1N1, H1N2, and H3N2 (3). An effective vaccination is recommended to reduce the disease burden in the swine population caused by SwIAV and its transmission to humans (4). The development of a universal influenza vaccine capable of eliciting cross-protective antibodies remains one of the top priorities globally. While neutralizing antibodies (nAbs) can block the infection and reduce the influenza virus replication, desired levels of its titer against variant strains of virus have not yet been achieved by existing vaccines. Despite the fact that non-nAbs (nNAbs) can bind viruses, they are unable to prevent infection in the cell culture system, and such antibodies are documented in influenza, rotavirus, HIV, cytomegalovirus, and SARS-CoV-2 infections (5). Interestingly, the role of nNAbs *in vivo* in protection against viruses has not been widely appreciated by the scientific community due to the lack of optimized immunological tools, especially in pigs and other large animal species, unlike in rodent models and humans. However, increasing evidence suggests that the induction of virus-specific nNAbs is an important correlation of protection (4). While nNAbs cannot prevent the entry of viruses into their host cell, they facilitate the recruitment of effector proteins or immune cells that can destroy the immune complex containing virus (4). Several nNAbs also mediate their effector function intracellularly either by direct blocking of the virus replication or by recruiting the Fc receptor expressing phagocytic cells (5).

Hemagglutinin inhibition (HAI) and virus neutralization (VN) assays are commonly employed for the detection and quantification of influenza-specific antibodies following infection or vaccination. Generally, HAI antibody titers are accepted as proxy for VN antibodies and are widely used to assess the efficacy of influenza vaccines (6). However, limited application of HAI assay has been reported, pointing the relevance of some non-HAI-mediated process as additional correlates of protection (7). The HA glycoprotein of influenza virus is composed of two domains: variable globular head as immunodominant receptor binding domain (RBD) and conserved stalk domain. The IAV-specific NAbs are typically directed to epitopes located in and around the viral RBD preventing virus binding to the target cells (8). Hence, mismatch between circulating strains of influenza results in compromised neutralization; however, it is likely that nNAbs and non-HAI antibodies might play a crucial role in protection in vaccine-mediated immune response (9, 10). Effector functions of the HA-stalk-specific nNAbs includes antibody-dependent phagocytosis (ADP) (11, 12), antibody-dependent cellular cytotoxicity (ADCC) (13), and antibody-mediated complement-dependent cell cytotoxicity (CDC), which may be critical in broader cross-protection against influenza (14–16). All these effector functions of nNAbs and non-HAI antibodies are mediated by Fcy receptors, contributing to protective immunity against influenza and other viral infections (17–19). Mostly, the conserved regions of influenza virus surface proteins are target for nNAbs, hence partially overcoming the emergence of NAbs escape mutants mediated by antigenic drift and antigenic shift in IAV (20).

NK cells are an important component of innate immunity during influenza virus infection because of their direct involvement in viral clearance (21, 22). NK cells mediate ADCC effector function by binding the antigen-bound IgG via FcyRIII (CD16). This results in the activation of NK cells and, consequently, the degranulation process to release lytic granules (perforin/granzymes) as well as secretion of antiviral cytokines such as tumor necrosis factor alpha (TNFα) and interferon gamma (IFNy). The role of the complement system (both classical and alternative pathways) is also critical in influenza virus infection or vaccination. Virion opsonization and its efficient lysis mediated by nNAbs through the complement system induced in response to infection or vaccination is another important humoral response against influenza (16).

Demonstration of the presence of influenza-specific ADCC and CDC antibodies was started in 1978 (23, 24). In mouse and human systems for measuring ADCC and CDC functions, we have optimized commercial kits. The antibody IgG1 isotype is involved in Fc‐effector functions of ADCC via FcγRIIIa expressed on macrophages and NK cells (25). The IgG1 isotype was shown to be superior to other isotypes, inducing ADCC as well as CDC functions (26). However, in the pig system, we do not have satisfactory tools to precisely delineate the IgG isotypes. Strategies to assess the role of cell-mediated immunity in response to SwIAV vaccines in pigs have already been demonstrated (27, 28); however, there have been limited studies and tools for estimating the SwIAV-specific ADCC and CDC antibodies in pigs. Therefore, developing and validating the ADCC and CDC assays in pig system is important.

Studies have shown that maternally derived antibody (MDA)-positive piglets have interference in induction of immunity following intramuscular SwIAV vaccination (29–32). In this study, we optimized the lactate dehydrogenase (LDH) release assay for the detection of pig HA-specific antibodies having ADCC and CDC activity. We validated the assays using serum samples archived from our previous two intranasal administered monovalent SwIAV nanoparticle-based vaccine trials, including a commercial multivalent SwIAV vaccine as control, conducted in both specific pathogen (influenza)-free and MDA-positive pigs (33, 34). Furthermore, when the ADCC and CDC data with VN, HAI, and antibody avidity data were compared, we did find an overall positive trend among all the different functional antibodies in pigs following vaccination and influenza infection. However, preexisting influenza-specific MDA strongly interfered with the generation of ADCC, but not CDC antibodies to inactivated whole SwIAV vaccines in pigs. In conclusion, we optimized the ADCC and CDC antibody functions specific to SwIAV in pigs, validated the assays, and revealed the trends among different antibody functions to influenza vaccination and infection in pigs.

## Material and methods

### Generation of MDCK cells stably expressing HA

**Figure 1:**
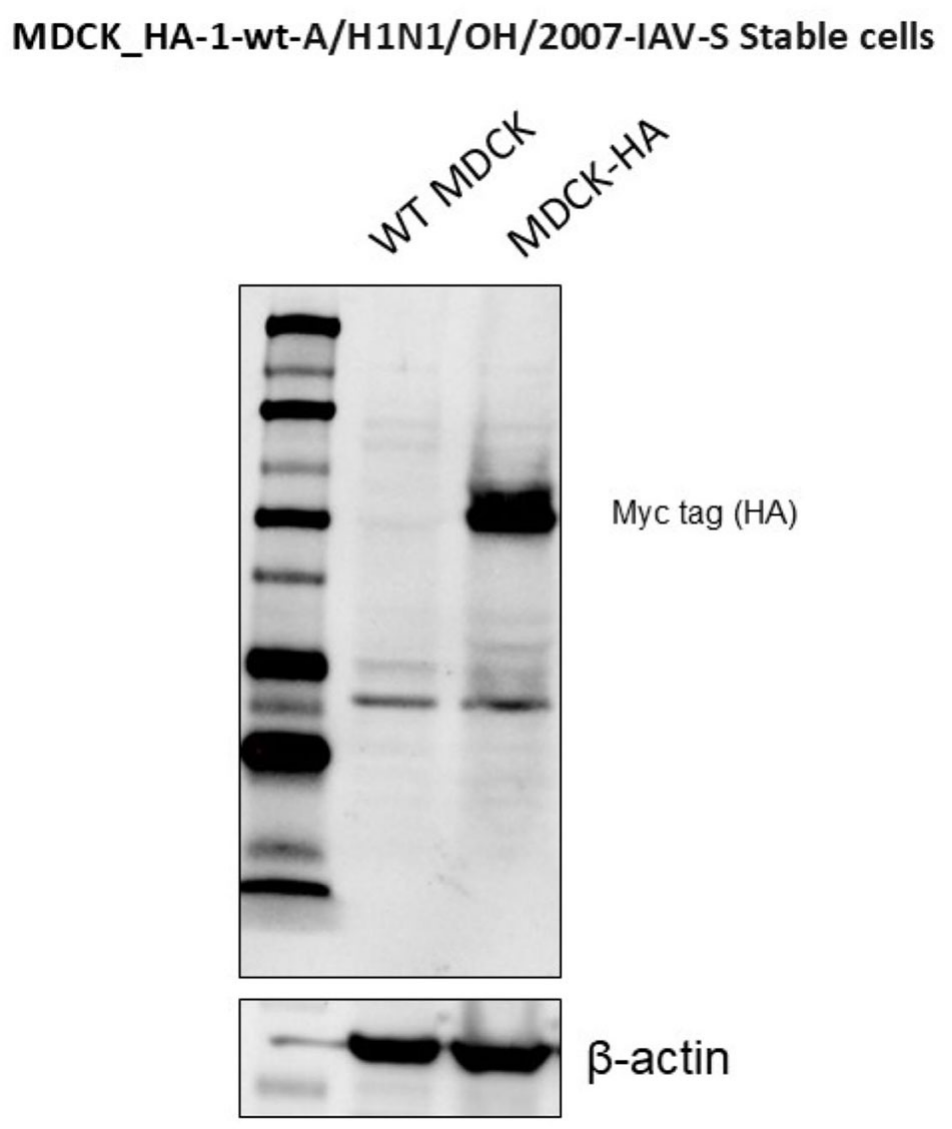
Stable expression of HA protein in transfected MDCK cells was detected throughout at different passages by Western blot.

To generate stable MDCK cells expressing HA from the SwIAV A/H1N1/OH/2007 strain, the full-length HA sequence was modified by replacing its transmembrane domain with that of PDGFRβ and adding a Myc-tag epitope to the 3' end. The PCR amplification of the HA region from the parental SwIAV A/H1N1/OH/2007 virus was performed using the following primers: forward primer 5'-ACTGCGGATCCATGAAGGCCATCCTGGTGGTGCTGCTGTACACCTTCACCA and reverse primer 3'-ACTGCCTCGAGCGGCCGCCTAACGTGGCTTCTTCT (1,808 bp). The amplified HA construct was then cloned into the lentiviral vector, pScalps (Addgene plasmid 99636, Watertown, Massachusetts, USA) (35) via enzymatic digestion with BamHI and XhoI (New England Biolabs, Ipswich, MA, USA), which corresponded to restriction sites included in the forward and reverse primers, respectively. Successful integration of the HA gene was confirmed by plasmid digestion followed by 1% agarose gel electrophoresis and detection of the Myc-tag epitope in transfected HEK 293T cells by immunofluorescence assay (IFA) (data not shown) and Western Blot (**Figure 1**) using an anti-Myc-tag antibody (9B11, Cell Signaling Technology, Danvers, MA, USA).

HEK 293T cells were cultured in complete media comprising Minimum Essential Medium (MEM) supplemented with 10% heat-inactivated fetal bovine serum (FBS), 2 mM L-glutamine, and antibiotics (penicillin, streptomycin, and gentamicin) at a density of 4 × 10^5^ cells per well in a six-well plate. After 24 h, the media was replaced, and cells were transfected with 1,000 ng of the generated lentiviral transfer plasmid, 750 ng of the human immunodeficiency virus (HIV) lentiviral packaging plasmid psPAX, and 1,000 ng of the VSV-G-encoding plasmid pMD2.G, using the Lipofectamine 3000 transfection reagent (Life Technologies, Carlsbad, CA, USA). Cell supernatants were harvested at 72 h post-transfection, centrifuged, and used for cell transduction.

MDCK cells were cultured in complete media at a similar density in a six-well plate. After 24 h, the media was removed, and cells were transduced with 500 µL of the lentiviral preparation per well. Control non-transduced cells received 500 µL of MEM cultured either with or without puromycin selection. During the 2-h adsorption period at 37°C, plates were gently tilted every 15–20 min. Subsequently, 1.5 mL of fresh complete media was added to each well, and cells were incubated at 37°C for 48 h. A puromycin kill curve was performed to determine the optimal concentration for selection, with concentrations ranging from 0 to 10 µg/mL. The higher concentration following the one causing 100% cell death was selected for generating stable cells. After 48 h of incubation, puromycin selection was initiated and continued until stable MDCK-HA cells were established.

### Antibody-dependent cellular cytotoxicity

Stably transfected MDCK-HA cells were used as target cells for the ADCC assay. The assay was performed as described previously with several modifications (36). Briefly, 8,000 MDCK-HA cells per well were seeded in round bottom 96-well plates overnight at 37°C with 5% CO_2_. Cells were washed thrice with 1× phosphate buffered saline (PBS) and incubated with heat-inactivated serum samples (1:25 dilution) for 30 min at 37°C. Following three washings with 1× PBS, freshly isolated pig peripheral blood mononuclear cells (PBMCs) (source of effector cells) from healthy slaughtered adult pigs were added in pre-titrated 80:1 effector-to-target ratio (E:T) to the round bottom 96-well plates and incubated for 4 h at 37°C with 5% CO_2_. Both the target and effector cells were cultured in serum-free AIM-V medium containing 5% Immune Cell Serum Replacement (Life Technologies, UK) along with 100 IU/mL penicillin and 100 µg/mL streptomycin. Following incubation, 50 μL of cell-free supernatant was collected and transferred into a 96-well flat bottom plate and the LDH release is measured using the Cytotox 96 non-radioactive cytotoxicity assay kit (Promega, Leiden, Netherlands) according to the manufacturer’s instructions. The absorbance was measured at an optical density (OD) of 490 nm in a plate reader. The ADCC activity of serum samples was assessed using the following formula: [corrected experimental OD_490_ (experimental sample with target cells and PBMC) − corrected effector spontaneous OD_490_ (PBMC spontaneous release without target cells) − corrected target spontaneous OD_490_ (target spontaneous release without effector cells)] divided by [corrected target maximum OD_490_ (maximum release of target cells in the presence of 0.8% Triton^®^ X-100) − corrected target spontaneous OD_490_ (target spontaneous release without effector cells)] × 100. We optimized the dilution of serum 1:25 to treat target cells, which provided the least background with high specific ADCC activity.

### Complement-dependent cell cytotoxicity

The CDC was determined as described previously with slight modifications (37). Briefly, 30,000 MDCK-HA cells per well were seeded in round bottom 96-well plates overnight at 37°C with 5% CO_2_. Subsequently, cells were washed thrice with 1× PBS and incubated with heat-inactivated serum samples at pre-titrated 1:25 dilution in serum-free AIM-V medium (Life Technologies, UK) containing 100 IU/mL penicillin and 100 µg/mL streptomycin for 30 min at 37°C. Following three washes with 1× PBS, 1:10 diluted Low-Tox rabbit complement (Cedarlane, Burlington, Ontario, Canada) in serum-free AIM-V medium was added, and the plate was incubated for 2 h at 37°C with 5% CO_2_. Subsequently, 50 μL of cell-free supernatant was transferred into a 96-well flat bottom plate and complement-dependent lysis of target cells was determined by measuring the LDH release using the Cytotox 96 nonradioactive cytotoxicity assay kit (Promega, Leiden, Netherlands) according to the manufacturer’s recommendations. The percentage specific lysis mediated by complement with serum samples was assessed using the following formula: (% lysis of target cells by antibody and complement − % lysis of target cells by complement alone)/(% maximum lysis of target cells in the presence of 0.8% Triton^®^ X-100 − % lysis of target cells by complement alone) × 100.

### Pig samples used to validate the assays

To validate our developed and optimized ADCC and CDC assays, we used sera samples from our earlier two vaccine trials in pigs with intranasal chitosan nanoparticle-based SwIAV vaccines or a commercial intramuscular administered multivalent SwIAV (FluSure XP^®^, Zoetis) vaccine (33, 34). The vaccines and related information are summarized in **Table 1** and **Supplementary Table 1**.

**Table 1:** Experiment#1: Conventional maternal antibody positive weaned piglets were vaccinated with mannose-conjugated or -unconjugated chitosan nanoparticle-based inactivated monovalent whole H1N2 SwIAV nanovaccines (Chit/mChit-SwIAV-NPs) and were challenged with the SwIAV H1N1-OH7 (A/Swine/OH/24366/2007) which has over 77% HA gene identity with the vaccine strain (H1N2 SwIAV).

Table 1 Experiment #1																				
	Serum IgG				Nasal Swab sIgA				BAL Fluid (IgG)		Lung Lysate (IgG)		HAI titers (log_2_)		Serum VNT titers (log_10_)		Serum ADCC (%)		Serum CDC (%)	
	ELISA OD_450_ (@ 1:100 dilution)		Avidity Index (@2.5M NH4HCN)		ELISA OD_450_ @ 1:8dilution		Avidity Index @0.625M NH4HCN		ELISA OD_450_ @ 1:50 dilution	Avidity Index @1.25M NH4HCN	ELISA OD_450_ @ 1:50 dilution	Avidity Index @1.25M NH4HCN	Serum	BAL						
	DPC0	DPC6	DPC0	DPC6	DPC0	DPC6	DPC0	DPC6	DPC6	DPC6	DPC6	DPC6	DPC6	DPC6	DPC0	DPC6	DPC0	DPC6	DPC0	DPC6
Mock	2.25 ± 0.07	1.87 ± 0.16	78.0 ± 0.06	67.0 ± 0.13	0.10 ± 0.06	0.10 ± 0.06	93.0 ± 0.07	71.0 ± 0.10	0.20 ± 0.05	49.0 ± 0.11	0.35 ± 0.16	53.0 ± 0.04	6.0 ± 0.23	4.0 ± 0.30	0.67 ± 0.39	1.20 ± 0.16	1.0 ± 0.33	6.0 ± 1.48	2.0 ± 0.65	2.0 ± 0.65
Mock Challenge	2.14 ± 0.22	1.86 ± 0.41	70.0 ± 0.23	58.0 ± 0.28	0.15 ± 0.26	0.15 ± 0.26	87.0 ± 0.11	64.0 ± 0.27	0.35 ± 0.38	49.0 ± 0.28	0.76 ± 0.51	28.0 ± 0.32	7.0 ± 0.19	4.0 ± 0.68	0.98 ± 0.46	1.98 ± 0.21	3.0 ± 0.45	24.0 ± 2.0	4.0 ± 1.28	5.0 ± 1.24
Commercial vaccine	2.13 ± 0.17	2.59 ± 0.04	74.0 ± 0.17	79.0 ± 0.09	0.46 ± 0.58	0.46 ± 0.58	90.0 ± 0.10	79.0 ± 0.15	1.20 ± 1.01	41.0 ± 0.20	2.23 ± 0.25	56.0 ± 0.39	9.0 ± 0.20	3.0 ± 0.48	2.13 ± 0.26	2.43 ± 0.10	4.0 ± 0.47	55.0 ± 0.86	52.0 ± 1.91	37.0 ± 2.17
Chit-SwIAV-NPs	1.96 ± 0.14	2.45 ± 0.05	71.0 ± 0.15	65.0 ± 0.22	0.81 ± 0.91	0.81 ± 0.91	85.0 ± 0.05	66.0 ± 0.26	0.99 ± 0.11	29.0 ± 0.17	2.18 ± 0.24	25.0 ± 0.26	7.0 ± 0.19	4.0 ± 0.24	1.08 ± 0.15	1.60 ± 0.53	4.0 ± 1.18	62.0 ± 2.26	12.0 ± 1.69	10.0 ± 1.48
mChit-SwIAV-NPs	2.09 ± 0.15	2.42 ± 0.04	73.0 ± 0.22	72.0 ± 0.02	0.93 ± 0.48	0.93 ± 0.48	93.0 ± 0.07	50.0 ± 0.14	1.30 ± 0.15	19.0 ± 0.06	2.19 ± 0.15	24.0 ± 0.21	7.0 ± 0.22	3.0 ± 0.90	1.15 ± 0.16	1.60 ± 0.24	6.0 ± 1.38	60.0 ± 3.07	5.0 ± 1.22	5.0 ± 1.49

Table 1 Experiment #2																				
	Serum IgG				Nasal Swab sIgA				BAL Fluid (IgG)		Lung Lysate (IgG)		HAI titers (log_2_) (34)		Serum VNT titers (log_10_) (34)		Serum ADCC (%)		Serum CDC (%)	
	ELISA OD_450_ @ 1:500 Dilution		Avidity Index @1.25M NH4HCN (34)		ELISA OD_450_ @ 1:4dilution		Avidity Index @1.25M NH4HCN (34)		ELISA OD_450_ @1:50 dilution	Avidity Index @1.25M NH4HCN (34)	ELISA OD_450_ @ 1:50 dilution	Avidity Index @1.25M NH4HCN (34)	Serum	BAL						
	DPC0	DPC6	DPC0	DPC6	DPC0	DPC6	DPC0	DPC6	DPC6	DPC6	DPC6	DPC6	DPC6	DPC6	DPC0	DPC6	DPC0	DPC6	DPC0	DPC6
Mock Challenge	0.14 ± 0.04	0.60 ± 0.34	63.0 ± 0.29	56.0 ± 0.18	0.09 ± 0.08	0.20 ± 0.17	79.0 ± 0.19	68.0 ± 0.34	0.080±0.14	75.0 ± 0.14	0.20 ± 0.39	67.0 ± 0.17	4.0 ± 0.61	2.0 ± 0.86	0.17 ± 0.07	1.5 ± 0.13	0.17 ± 0.0	0.0 ± 0.0	25.0 ± 4.57	22.0 ± 3.29
Commercial vaccine	0.58 ± 0.37	0.82 ± 0.44	27.0 ± 0.25	43.0 ± 0.26	0.08 ± 0.07	0.74 ± 0.54	81.0 ± 0.14	50.0 ± 0.18	0.56 ± 0.12	47.0 ± 0.47	1.24 ± 0.17	60.0 ± 0.34	7.0 ± 0.20	1.0 ± 0.47	1.43 ± 0.25	2.24 ± 0.14	40.0 ± 5.56	35.0 ± 3.06	25.0 ± 3.86	32.0 ± 4.98
mChit-SwIAV+S100-**e**NPs	0.19 ± 0.10	0.45 ± 0.12	49.0 ± 0.19	39.0 ± 0.28	0.08 ± 0.05	0.43 ± 0.32	82.0 ± 0.15	41.0 ± 0.18	0.55 ± 0.19	34.0 ± 0.19	0.79 ± 0.33	58.0 ± 0.15	5.0 ± 0.22	2.0 ± 0.34	0.0 ± 0.0	1.85 ± 0.09	30.0 ± 3.38	15.0 ± 1.96	54.0 ± 4.90	52.0 ± 2.92
mChit-SwIAV+S100-**s**NPs	0.81 ± 0.72	0.67 ± 0.40	21.0 ± 0.30	48.0 ± 0.20	0.22 ± 0.47	0.55 ± 0.46	55.0 ± 0.49	43.0 ± 0.24	1.26 ± 0.22	62.0 ± 0.62	1.23 ± 0.11	48.0 ± 0.12	5.0 ± 0.45	4.0 ± 0.21	0.0 ± 0.0	1.7 ± 0.12	31.0 ± 3.87	29.0 ± 8.48	24.0 ± 3.95	33.0 ± 4.99

Experiment#2: Specific pathogen (influenza) free piglets were vaccinated with monovalent whole H1N2 SwIAV antigen and STING agonist (S100) adjuvant either encapsulated (mChit-SwIAV+S100-**e**NPs) in mannose-conjugated chitosan nanoparticle or surface adsorbed (mChit-SwIAV+S100-**s**NPs) on mannose-conjugated chitosan nanoparticle, and challenged with the pandemic CA09-H1N1 [A/California/04/2009 (H1N1)] which has over 78% HA gene identity with the vaccine strain (H1N2 SwIAV). Samples of serum, BAL fluid, nasal swab, and lung lysate collected at DPC0 and DPC6 were subjected to immunological assays to detect the challenge IAV specific IgG, IgA, antibody avidity index, HAI and VN tiers and ADCC and CDC activity. Commercial intramuscular multivalent SwIAV (FluSure XP®, Zoetis) vaccine was administered per the manufacturer’s recommendation. Each data is an average of 3-4 pigs +/- SEM for Experiment#1 and average of 5-6 pigs +/- SEM for Experiment#2.

### Virus neutralization assay

Virus nAb titers were determined as described previously with slight modifications (38). Pig sera collected at both DPC 0 and 6 were subjected to heat inactivation for 30 min at 56°C. Briefly, a twofold serial dilution (starting from 1:5) of respective serum samples was mixed with an equal volume of SwIAV H1N1/OH/2007 (100 TCID_50_/well) in a 96-well round-bottom plate and incubated at 37°C in 5% CO_2_ for 1.5 h. One hundred microliters of sample and virus mix was transferred onto pre-adhered MDCK cells monolayer of over 90% confluency and incubated at 37°C in 5% CO_2_ for 1.5 h before adding additional 100 µL/well 1× DMEM containing TPCK-treated trypsin (2.0 µg/mL), and incubated for 44 h. The neutralizing activity of each serum sample was recorded as the reciprocal of the highest dilution showing complete inhibition of virus infection in MDCK cells assessed by fluorescent antibody test. Finally, the virus nAb titers were transformed to log_10_ values for comparison between different experimental vaccine groups.

### Hemagglutination inhibition assay

The HAI titer specific to SwIAV H1N1/OH/2007 and 2009 pandemic CA09-H1N1 IAV in sera and bronchoalveolar lavage (BAL) fluid (**Table 1**) was determined as described previously (39). Briefly, heat-inactivated serially 2-fold diluted BAL fluid samples were mixed with 8 HAU of SwIAV (H1N1/OH/2007) in 50 µL and incubated for 1 h at 37°C. The HAI titers were determined as the reciprocal of the highest dilution of the samples that completely prevented the hemagglutination of 1% turkey red blood cells. The HAI titers were transformed into log_2_ values for comparison between different experimental groups. The HAI titers in serum and BAL fluid samples were reported earlier (33, 34) and are mentioned in **Table 1** for comparison purposes.

### Avidity of virus-specific antibodies

Determination of influenza virus-specific antibody avidity present in serum, BAL fluid, lung lysate, and nasal specimens collected from experimental animals was performed by an avidity enzyme-linked immunosorbent assay (ELISA) as described earlier (40). The dilutions of samples:serum (1:100), BAL fluid (1:50), lung lysate (1:50), and nasal swab specimens (1:8) were used in the assay. Briefly, the 96-well ELISA plates were adsorbed with pre-titrated inactivated SwIAV antigen (H1N1/OH/2007) and the procedure followed the same steps of ELISA as described earlier with an additional step of incubating the plate with a chaotropic agent, ammonium thiocyanate (NH_4_SCN), at different indicated molar concentrations (0 to 5 M) for 15 min at room temperature following treatment with test samples. The antibody avidity index was calculated as mean OD_450_ (NH_4_SCN-treated samples)/mean OD_450_ (PBST-treated samples) and multiplied by 100. The antibody avidity data in serum, BAL fluid, lung lysate, and nasal swab specimens in Experiment #2 (and not Experiment #1) were already reported (34) and are mentioned in **Table 1** for comparison purposes.

### Statistical analysis

Statistical differences between multi-group comparisons were performed using one-way ANOVA or two-way ANOVA followed by Tukey’s multiple comparison test in Prism 10 (GraphPad Software, Inc., CA, USA). The group data are presented as the mean ± SEM of three to four pigs (Experiment #1) and six to seven pigs (Experiment # 2) with a statistical significance of *p* < 0.05. A box-and-whisker plot shows interquartile ranges, and horizontal lines show group median.

## Results

### Stable expression of SwIAV-HA in MDCK cells

Expression of SwIAV H1N1 HA protein in stably transfected MDCK cells was confirmed by the indirect IFA on both permeabilized and non-permeabilized cells (data not shown), and by WB analysis detected in transfected MDCK cells at different passages using the anti-Myc-tag antibody (9B11, Cell Signaling Technology, Danvers, MA, USA) (**Figure 1**). Non-transduced MDCK cells served as negative control, while β-actin served as a loading control in the WB analysis.

### Optimization of SwIAV-specific ADCC activity

**Figure 2:**
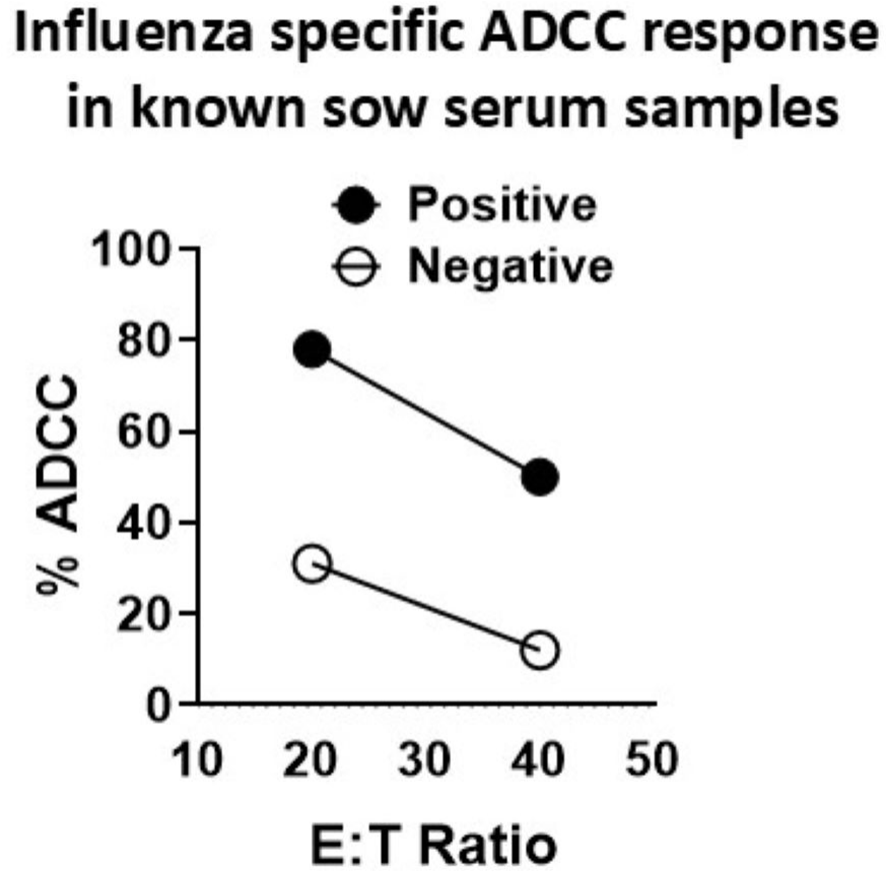
Optimization of influenza A virus-specific ADCC activity in the serum of adult vaccinated pigs. Serum samples collected from sows vaccinated (positive) or unvaccinated (negative) twice with a commercial swine influenza multivalent vaccine were used to optimize the ADCC activity measured by LDH release assay. Freshly isolated pig peripheral blood mononuclear cells (PBMCs) from healthy slaughtered pigs served as effector cells and the MDCK-HA cells served as targets, which were pretreated with SwIAV-specific positive or -negative sera at 1:25 dilution for 4 h at 37°C, and the effector-to-target ratios (E:T) used in the assay were 20:1 and 40:1. Each marking on a line graph represents the % lysis of target cells treated with a known influenza antibody-positive or -negative serum to determine the virus-specific ADCC activity.

We optimized the conditions such as temperature and duration of incubation of pig serum-treated target cells with effectors to measure the ADCC activity in pigs. In an earlier study, influenza-specific ADCC antibodies measured in pigs had few limitations. In that study, H1N1-specific pig immune serum was diluted 1:10 and detected a maximum 10% ADCC activity over the high background activity of greater than 15% in negative sera-treated MDCK-HA target cells, used at an E:T ratio of 20:1, with healthy pig PBMC as effectors (36). We made use of serum samples collected from unvaccinated sows (negative) and sows vaccinated with a commercial SwIAV multivalent (FluSure XP^®^) vaccine to optimize the ADCC assay. We specifically determined the ratio of effector pig PBMC to target MDCK-HA cells that allowed the detection of ADCC activity with high specificity and the least background. The target MDCK-HA cells were pre-incubated with sera at 1:25 dilution for 4 h at 37°C and then co-cultured with effector cells. Under these experimental conditions, sera from unvaccinated sows showed some background ADCC activity at an E:T of 20:1 and 40:1 (**Figure 2**), while the sera of vaccinated sows exhibited ADCC activity of 50% and 80% at a target ratio of 40:1 and 20:1, respectively, which was significantly higher than the background ADCC signals of 15% and 25% induced by sera from unvaccinated sows (**Figure 2**). Therefore, the E:T of 80:1 was used in subsequent studies.

### Optimization of SwIAV-specific CDC activity

Serum samples collected from unvaccinated sows (negative) or sows vaccinated with a commercial SwIAV multivalent (FluSure XP^®^) vaccine were used to optimize the CDC assay. Similar to ADCC, serum dilution was optimized at 1:25 in serum-free AIM-V medium, which resulted in high signal and low background values. The 10% Low Tox rabbit complement provided the least background with high specific CDC activity when incubated at 37°C for 2 h in the assay (**Tables 2**–**4**).

**Table 2:** Optimization of ideal serum dilution and incubation temperature for CDC assay.

37 ^0^C X 30 min incubation					4 ^0^C X 30 min incubation			
Serum dilution	1:25	1:50	1:100	1:200	1:25	1:50	1:100	1:200
CDC with positive serum	37	10	1	4	20	11	7	1
CDC with negative Serum	0.4	2	0	4	2	0	0	0

Monolayer of MDCK-HA cells were incubated with heat-inactivated SwIAV positive or negative serum at indicated dilutions for 30 min at either 37^0^C or 4^0^C. Followed by Low-Tox rabbit complement (10%) in serum-free AIM-V medium was added and the plate was incubated. The harvested cell-free supernatant was measured for the complement-dependent lysis of target cells by measuring the LDH release using the nonradioactive cytotoxicity assay kit.

**Table 3:** Optimization of ideal incubation time for CDC assay.

Incubation time	37 ^0^C 2h	37 ^0^C 4h
CDC with positive serum (1:25 dilution)	52	45
CDC with negative Serum (1:25 dilution)	0	0

Monolayer of MDCK-HA cells were incubated with heat-inactivated SwIAV positive or negative serum at a dilution 1:25 for either 2 or 4 hours, and Low-Tox rabbit complement diluted (10%) in serum-free AIM-V medium was added and the plate was incubated. The harvested cell-free supernatant was measured for the complement-dependent lysis of target cells by measuring the LDH release using the nonradioactive cytotoxicity assay kit.

**Table 4:** Optimization of ideal complement concentration (5%, 10% & 20%) for CDC assay.

Complement concentration	5%	10%	20%
CDC with positive serum (1:25 dilution)	41	60	72
CDC with negative Serum (1:25 dilution)	13	14	25

Monolayer of MDCK-HA cells were incubated with heat-inactivated SwIAV positive or negative serum at a dilution 1:25 for 30 min and incubated at 37^0^C, and Low-Tox rabbit complement at 5%, 10% or 20% concentration diluted in serum-free AIM-V medium was added and the plate was incubated. The harvested cell-free supernatant was measured for the complement-dependent lysis of target cells by measuring the LDH release using nonradioactive cytotoxicity assay kit.

### Application of optimized ADCC and CDC assays to characterize antibody functions in intranasal vaccinated pigs

**Figure 3:**
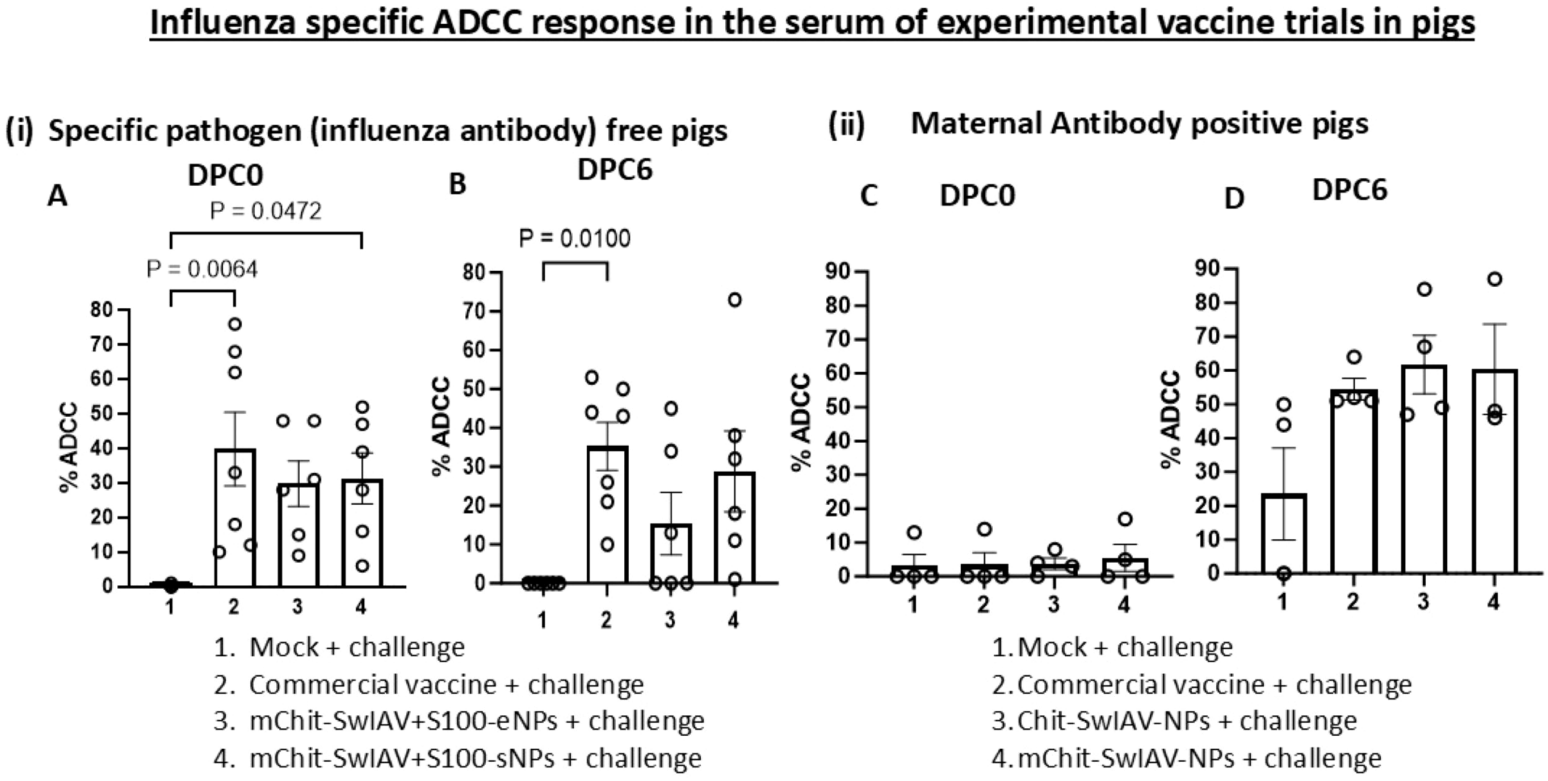
Influenza A virus-specific ADCC activity in the serum of experimental SwIAV vaccinated pigs. (i) Specific pathogen-free (6–7 per group) pigs and (ii) maternal antibody-positive (3–4 per group) pigs at 4–5 weeks of age were prime-boost vaccinated at 3-week intervals intranasally with Chit-SwIAV-NPs, mChit-SwIAV-NPs, mChit-SwIAV+S100-eNPs, and mChit-SwIAV+S100-sNPs, or intramuscularly with the commercial FluSure XP^®^ vaccine and challenged with a heterologous **(A, B)** 2009 pandemic H1N1 IAV and **(C, D)** H1N1/OH/2007 SwIAV. Sera collected at **(A, C)** DPC0 and **(B, D)** DPC6 from different vaccinated pigs were used to pretreat the target cells at 1:25 dilution as mentioned in the **Figure 2** legend. Freshly isolated PBMCs from healthy slaughtered pigs served as effectors used at an E:T ratio of 80:1 in the ADCC assay. Each marking on a bar represents the % lysis of target cells by individual pig serum and each bar is the mean ± SEM of each vaccine group. One-way ANOVA with Tukey’s multiple comparisons *post-hoc* test was employed to determine the *p*-values between virus-challenged vaccinated versus mock groups (*p* < 0.05).

In vaccinated and virus-challenged pig serum samples (diluted 1:25), the optimum E:T ratio of 80:1 was found ideal, because we could achieve over 60% target ADCC lysis with less than 15% background activity in the assay (**Figure 3**). To validate the ADCC and CDC assays, we measured SwIAV-specific ADCC activity in the serum of pigs previously immunized with four different nanovaccine formulations and a commercial swine flu vaccine, while in influenza antibody-free pigs vaccinated with two types of nanovaccines containing a STING adjuvant, we observed relatively low levels of ADCC activity than the commercial SwIAV vaccine (**Figure 3(i) A, B**).

In weaned MDA-positive piglets, interference in the induction of immunity to intramuscular administered inactivated SwIAV vaccines was observed, leading to poor induction of specific antibody responses, which is associated with evidence of vaccine-associated enhanced respiratory disease (29–32). Therefore, in this study, we assayed for SwIAV-specific ADCC and CDC activity in MDA-positive pigs that received inactivated SwIAV vaccine and challenged. Our data indicated that in MDA-positive pigs administered with two types of nanovaccines and a commercial swine flu vaccine, the ADCC activity was not detectable post prime-boost vaccination before challenge at DPC0, while after a challenge infection at DPC6, we observed enhanced induction of ADCC function (**Figures 3(ii) C, D**). The levels of ADCC activity with both nanovaccines and commercial swine flu vaccinates were comparable (**Figures 3(ii) C, D**).

**Figure 4:**
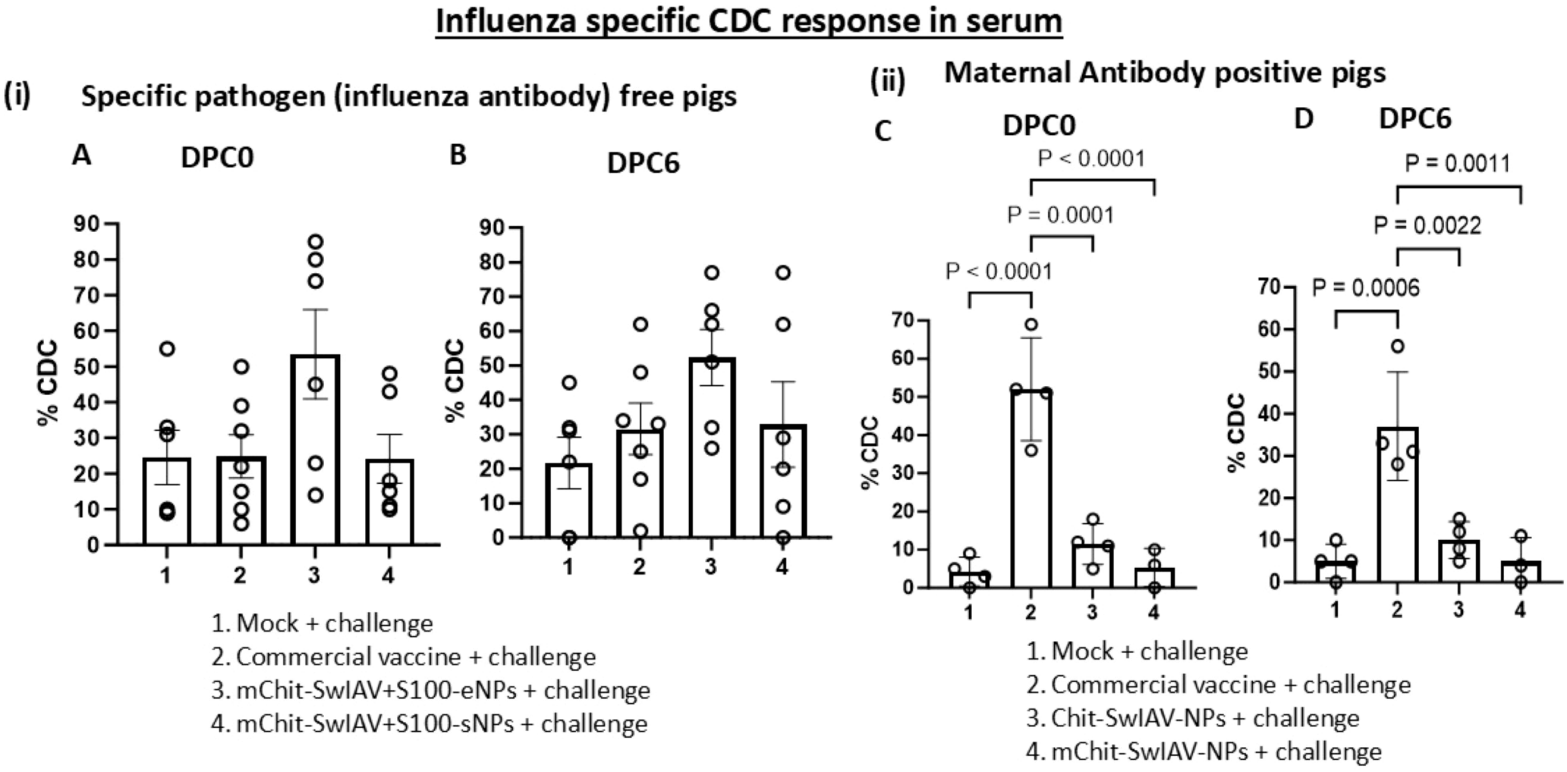
Influenza A virus-specific CDC activity in the serum of experimental SwIAV vaccinated pigs. (i) Specific pathogen-free (6–7 per group) and (ii) maternal antibody-positive (3–4 per group) pigs were prime-boost vaccinated and challenged with a heterologous SwIAV as described in the **Figure 3** legend. Sera collected at **(A, C)** DPC0 and **(B, D)** DPC6 from different vaccinated groups were used to pretreat the MDCK-HA target cells at 1:25 dilution as mentioned in the **Figure 2** legend in the presence of rabbit complement. The CDC-mediated killing (% lysis of target cells) was measured in duplicates by LDH release assay. Each marking on a bar represents the % lysis of target cells by individual pig serum, and each bar is the mean ± SEM. One-way ANOVA with Tukey’s multiple comparisons *post-hoc* test was employed to determine the *p*-values between virus-challenged vaccinated versus mock groups (*p* < 0.05).

In influenza antibody-free pigs vaccinated with two other types of nanovaccines containing a STING adjuvant (34), one of the nanovaccines containing whole inactivated SwIAV with STING adjuvant entrapped in NPs induced enhanced specific CDC activity compared to the cohort groups that received NP surface adsorbed vaccine cargo with adjuvant formulation and the commercial swine flu vaccine (**Figures 4(i) A, B**). The MDA positive pigs from commercial vaccine group displayed a significantly (*p* < 0.01) higher CDC activity as compared to both the nanovaccines group and mock challenge group (**Figures 4(ii) C, D**). Interestingly, the CDC activity did not increase further after the challenge infection at DPC6 compared to that at DPC0 in both vaccine trials (**Figure 4i, ii**).

### Virus neutralizing and hemagglutination inhibition antibody titers in vaccinated pigs

**Figure 5:**
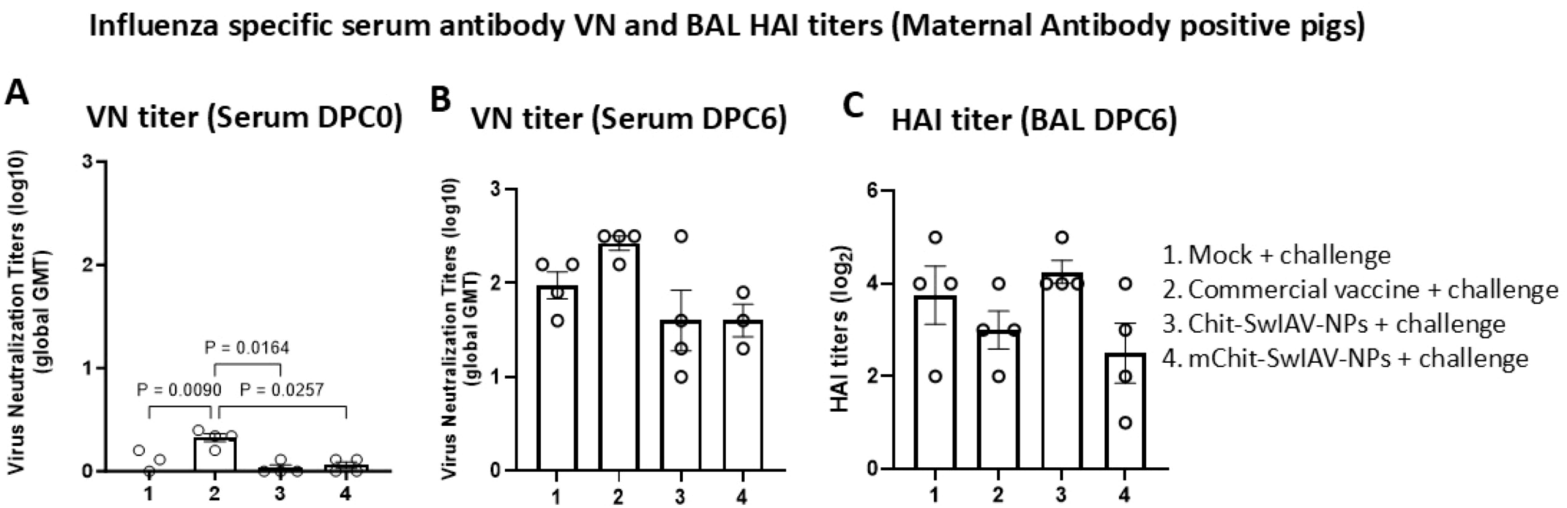
Influenza A virus-specific virus neutralization (VN) and hemagglutination inhibition (HAI) antibody titers in the serum and BAL fluid of experimental pigs, respectively. Maternal antibody-positive weaned 4-week-old pigs were prime-boost vaccinated at 3-week intervals intranasally with Chit-SwIAV-NPs and mChit-SwIAV-NPs vaccine or a commercial FluSure XP^®^ vaccine intramuscularly and challenged with a heterologous H1N1/OH/2007 SwIAV. **(A)** VN titers in serum at DPC0 and **(B)** DPC6 and **(C)** BAL fluid HAI endpoint titers at DPC6 were assessed against the challenge virus (H1N1/OH/2007). The VN titer is the reciprocal endpoint dilution transformed to a log_10_ value and plotted as a geometric mean titer (GMT) with SEM from triplicate wells. The HAI titers is the reciprocal endpoint dilution transformed to log_2_ values. Each marking on a bar represents the titer of an individual pig, and each bar is the average of three to four pigs (mean ± SEM). One-way ANOVA with Tukey’s multiple comparisons *post-hoc* test was employed to determine the *p*-values between groups (*p* < 0.05).

The virus neutralizing (VN) and HAI antibody titers in the serum samples of MDA-positive pigs that were vaccinated and challenged using the SwIAV H1N1-OH7 were determined. The lower detection limit of VN and HAI antibody titers were 1:5 and 1:2, respectively. At DPC0, only in the serum of the commercial vaccine group low levels of VN titers were detected, and the data were significantly (*p* < 0.05) higher compared to mock challenge and both the nanovaccine groups (**Figure 5A**). At DPC6, relatively higher VN titers were detected in the commercial vaccine compared to both the nanovaccines received pig groups (**Figure 5B**). Furthermore, no marked differences were observed in HAI titers in the BAL fluid of all the vaccinated and mock challenge pig groups (**Figure 5C**). It is evident that the commercial SwIAV vaccine is superior to nanovaccine candidates in eliciting relatively higher VN antibody titers in serum, but the same was not true for HAI titers in both the serum and BAL fluid (**Figures 5A, C**) (**Table 1**).

### SwIAV-specific antibodies (IgG and IgA) avidity in vaccinated pigs

**Figure 6:**
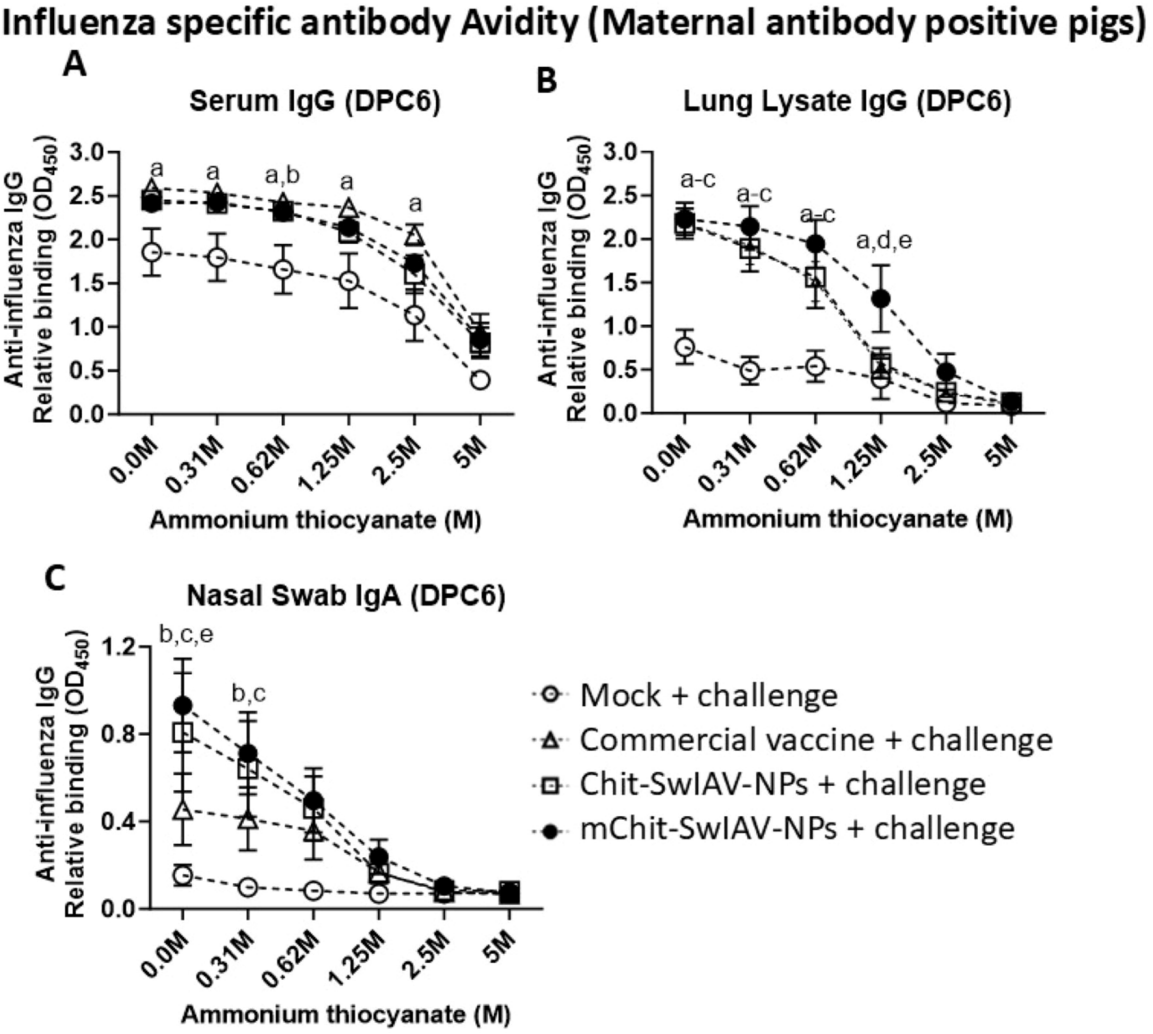
Influenza A virus-specific IgG and IgA antibody avidity in the serum, lung lysate, and nasal swab samples of experimental vaccinated pigs at different concentrations of ammonium thiocyanate (NH_4_SCN). Samples collected at DPC6 from the maternal antibody-positive weaned pigs were vaccinated and challenged as described in the **Figure 5** legend. Relative binding avidity of SwIAV-specific IgG in **(A)** serum (1:100 dilution), **(B)** lung lysate (1:50 dilution), and **(C)** IgA in nasal swab (1:8 dilution), performed both in the absence and in the presence of NH_4_SCN at various molar concentrations. Each marking represents the average OD value of three to four pigs (mean ± SEM). The statistical difference between experimental groups is considered as significant and denoted with the following lowercase letters: a, mock + challenge vs. commercial vaccine; b, mock + challenge vs. Chit-SwIAV-NPs; c, mock + challenge vs. mChit-SwIAV-NPs; d, commercial vaccine vs. Chit-SwIAV-NPs; and e, commercial vaccine vs. mChit-SwIAV-NPs. Two-way ANOVA with Tukey’s multiple comparisons *post-hoc* test was employed to determine the *p*-values between the groups (*p* < 0.05).

Avidity refers to strength of binding of antigen and antibody, and it is considered as an important correlate of protective immunity induced by vaccines. As expected, the OD_450_ values declined in all the vaccine groups with increasing concentrations of NH_4_SCN ranging from 0 to 5.0 M. To determine the ideal concentration of the chaotropic agent to induce dissociation of immune complexes, a nonlinear regression model was plotted using the OD_450_ values obtained at twofold dilution of NH_4_SCN (5.0, 2.5, 1.25, 0.625, and 0.31 M) against control (0 M). At 2.5 M of NH_4_SCN, SwIAV-specific serum IgGs were still bound to the antigen, while at 1.25 and 0.625 M of NH_4_SCN solution, the lung lysate antibodies and the nasal swab IgA antibodies, respectively, were still bound to the SwIAV antigen (**Figures 6A, C**). Avidity of specific IgG in serum was comparable in both the nanovaccines and commercial SwIAV vaccinates (**Figure 6A**). In the nanovaccine-administered maternal antibody-positive pig groups, lung lysate and nasal swab antibodies had a significantly higher specific antibody avidity in some of the NH_4_SCN concentrations than in the commercial vaccine and mock challenge groups (**Figures 6B, C**). The avidity data of influenza antibody free pigs are presented in **Table 1**.

## Discussion

The effectiveness of influenza vaccines is widely assessed by evaluating the humoral immune responses. In the process of development and validation of new broad-spectrum influenza vaccines, a mechanistic study to evaluate various types of functional antibodies—both virus neutralizing (NAbs) and non-neutralizing (nNAbs) through reliable methods for quantification in vaccinated animals—is important. This will help understand the correlates of protection, especially the cross-protection ability of vaccines. Therefore, measurement of different functional activities of antibodies including nNAbs should be prioritized in all the influenza vaccine preclinical and clinical trials, because both contribute significantly towards protection, leading to the development of novel influenza vaccination strategies directed towards boosting the quality of nNAbs. The effector functions of NAbs and nNAbs specific to influenza viruses are mediated through engagement of a constant region of antibodies (Fc), and it is not necessarily exclusive to nNAbs. Interestingly, it has been reported that NAbs specific to influenza and HIV elicit optimal function following their engagement with Fcy receptors (41, 42).

Both HA and neuraminidase (NA) proteins of influenza virus are considered as antigenic determinants for ADCC antibody (43). An anamnestic ADCC response has been observed following the IAV heterosubtypic challenge infection. Functional HA-specific antibodies capable of inducing ADCC appear before HAI antibodies and exhibit relatively higher breadth of binding to cognate antigen in comparison to NAbs (44). The ADCC-inducing HA-specific antibodies have been shown to reduce the severity of illness with increased virus clearance (45). In addition, increased ADCC activity positively correlates with robust HAI responses following vaccination (44). Even though HAI antibodies compete with ADCC antibodies to access the common antigenic epitopes (46), the role of ADCC in protection against influenza infection or vaccination remains elusive. Different animal models such as mouse, ferret, and non-human primates have been successfully used to assess the protective efficacy of ADCC-based vaccines (47, 48).

Despite numerous reports on NAbs and HAI antibodies induced by SwIAV vaccines, limited literature is available on vaccine-induced ADCC and CDC responses in pigs. The HA-targeting antibodies have been known to protect against influenza infections (49). Therefore, we used stably transfected MDCK cells engineered to express the full-length open reading frame of HA as target cells in both the ADCC and CDC assays and using the LDH release as a readout. The LDH release assay has been the most biologically relevant assay to determine the role of antibodies contributing to ADCC and CDC, as it directly measures the antibody-mediated killing of influenza-infected cells (50, 51). In human samples, the functional role of ADCC antibodies have been successfully demonstrated using target cells stably expressing HA protein (50). In pigs, this is the first report of detecting the ADCC and CDC antibodies induced in response to whole inactivated SwIAV vaccines (50). We propose that a blocking assay using monoclonal antibodies targeting specific HA domains could determine whether ADCC responses are primarily directed to the conserved HA stalk or more variable head regions.

Furthermore, validating the assays using samples of two relevant vaccine trials has provided a unique opportunity to quantify the ADCC and CDC antibodies. The generation of cross-reactive influenza-specific HAI-, VN-, ADCC-, and CDC-mediating antibodies is crucial following vaccination or infection. Our previous reports suggest that the SwIAV nanovaccines elicit cross-reactive NAbs and HAI antibodies against divergent viral strains, but we do not know the other functions of specific antibodies (33, 34). Therefore, our goal was to optimize the pig-specific ADCC and CDC assays and validate them using the archived samples of the two previous SwIAV nanovaccine trials (33, 34), determine the cross-reactive ADCC and CDC antibodies and correlate the data with specific VN and HAI titers and antibody avidity.

Our data indicated that there was a cross-reactive ADCC and CDC antibody activity, because the whole inactivated SwIAV H1N2 used in the nanovaccines was heterologous to both the challenge viruses (33, 34). The heterogeneity among the vaccine and challenge viruses was compared for HA gene identify by retrieving data of isolates from the GenBank public database (52) and using the BLAST server (53–56) (**Supplementary Table 2**). The commercial SwIAV multivalent vaccine has four SwIAV strains that belong to two H1N1, one H1N2, and one H3N2 subtype, and when compared among each other with both the challenge viruses, one of the H1N1 viruses in the vaccine has over 93% HA gene identity. Thus, the commercial swine flu vaccine and challenge viruses are considered homologous to each other.

Despite optimizing and validating the sensitivity of pig-specific ADCC and CDC assay using archived vaccinated piglets and sow serum samples, there are still some limitations such as high variability in the percentage cytotoxicity mediated by positive and negative serum samples within the treatment groups. Replacing the routine RPMI growth media with serum-free AIM-V medium containing 5% Immune Cell Serum Replacement for diluting serum samples and culturing of target and effector cells are critical for the assay, which is consistent with the previous report (57). Furthermore, ADCC and CDC assays were performed on sera only and not on BAL, nasal swabs, and lung lysate samples. Predominance of antibodies (IgG class) exhibiting ADCC and CDC activities in serum compared to other samples and the availability of limited samples (BAL, nasal swabs, and lung lysate) in our source directed us to perform these functional antibody assays using only serum.

We detected the cross-reactive HA-specific ADCC antibodies in pigs in response to nanovaccine candidates delivered intranasally. In the serum collected from influenza MDA-positive pigs at DPC0, the ADCC antibody was not induced, but upon challenge infection at DPC6, high levels of specific ADCC antibody were detected. In contrast, in influenza antibody-free pigs both at DPC0 and DPC6, high levels of virus-specific ADCC-mediating antibody production were detected, suggesting that in pigs having preexisting antibodies, there was a suppression in the induction of ADCC antibodies to an inactivated SwIAV vaccine, but the live virus exposure in vaccinates rescued that response. This pattern of induction of ADCC antibody is consistent with previous reports (58, 59). Interestingly, both the commercial swine flu and nanovaccines elicited high levels of specific ADCC antibody response, which may be associated with the identical HA subtype of the virus (H1) used in challenge and vaccine viruses, associated with the use of target MDCK cells expressing H1N1 HA. Our data revealed that MDA-positive pigs vaccinated intranasally with monovalent nanovaccines induced comparable levels of ADCC responses to that of a commercial swine flu vaccine, despite over 77% HA gene identify with the challenge virus. Furthermore, there are 13 amino acid differences in HA between vaccine and challenge viruses, which could have affected the functionality of ADCC antibodies (60). Hence, our findings support the previous observation that cross-reactive ADCC antibodies appear to confer a broad spectrum of protection and may contribute to the design of universal influenza vaccines in the future (59). In our study, maternal antibodies appear to suppress ADCC responses but not CDC responses, and we do not know the mechanistic explanation, which needs further investigation both in the pig and other animal models. It is possible that maternal antibodies neutralize the vaccine antigen prior to induction of ADCC-mediating antibodies, or if they happen to inhibit B-cell activation necessary for producing ADCC antibodies, the ADCC response can be consequently suppressed. Furthermore, the activation of pre-existing B cells may lead to class switching from IgG to non-ADCC isotypes (61).

The magnitude of ADCC based on serum IgG is, at least in part, influenced by the ratio of ADCC-inducing (nNAbs) and -inhibiting (NAbs) antibodies (62). Adjuvants may influence the pattern of antibody glycosylation generated by vaccines, thereby affecting the functionality of ADCC antibodies (63). Overall, the heightened specific ADCC antibody responses induced by intranasal delivered nanovaccines helped in the better clearance of the heterologous challenge virus in comparison to the commercial vaccine (33, 34), consistent with previous reports pointing to the cross-reactive and protective nature of ADCC-mediating antibodies in influenza infection/vaccination (45, 59, 64). The pre-existing IAV-specific neutralizing and HAI antibodies were reported to affect the potency of ADCC and the ADCC-based vaccine (46, 47). Therefore, the efficacy of the ADCC-based vaccine may be affected by pre-existing immunity (47). This is consistent with our data of not detecting any ADCC activity post-SwIAV vaccination.

The complement system can serve as a link between innate and adaptive immunity, and understanding its role in vaccine development is crucial. Under *in vitro* settings, the role of complement in the neutralization of the influenza virus using specific antibodies has been established (65, 66). Inclusion of the STING adjuvant in SwIAV nanovaccine formulation had a substantial effect on induction of specific CDC antibody reflected at both DPC0 and DPC6. The role of CDC has been investigated in other similar conditions such as HIV, hepatitis B, and herpes virus infections (13, 67). The major mechanism of CDC antibodies in virus clearance is through lysis of infected cells, thereby preventing maximal replication and release of infectious virions. Free virions released from infected cells can also be neutralized by specific CDC antibodies and subsequently removed by phagocytosis (68). The induction of specific CDC antibodies in MDA-positive pigs was not high at both DPC0 and DPC6 in nano-vaccinates compared to commercial vaccine, which elicited a robust CDC antibody response, suggesting the influence of route of vaccination (intramuscular versus intranasal), HA gene identity, and monovalent versus multivalent nature of the vaccines.

Antibody avidity indicates the priming of immunological memory, as vaccination results in antibody maturation and, hence, generation of antibodies with increased avidity. Antibody avidity is considered as a qualitative response index and can directly correlate with protection (69). Conversely, inadequate levels of avidity have been linked to antibody-mediated disease enhancement following pandemic influenza vaccinations (70). Virus-specific IgG avidity in serum was comparable in both commercial swine flu and nano-vaccinates, while the nasal passage known to be rich in sIgA showed significantly higher avidity compared to the commercial vaccine. In the lung lysate, which is known to possess both IgG and sIgA subclass antibodies, relatively higher antibody avidity in nano-vaccinates was observed compared to the commercial vaccine. This is important because both mucosal and systemic antibodies have been previously shown to be involved in protection against influenza infection (71). However, the immune response after vaccination may be influenced by several factors such as vaccine type and age of recipients. Our data in pigs vaccinated with a commercial multivalent vaccine and monovalent nanovaccines revealed a strong positive association among the NAbs, HAI, antibody avidity, ADCC, and CDC antibody activity (**Table 1**). This further highlights the importance of augmenting influenza specific both mucosal and systemic antibody responses using potent nasal vaccine delivery system like mucoadhesive chitosan nanoparticle platform for achieving the cross-protective immunity.

In conclusion, induction of ADCC response has been one of the important criteria for potential vaccine candidates by the World Health Organization. Therefore, understanding this response elicited by candidate vaccines irrespective of species may provide valuable insights into crucial immune correlates of protection. Together, our data provide evidence that both cross-reactive NAbs and nNAbs induced by vaccination could play an important role in broad-spectrum immunity against heterologous challenge influenza virus infection. Another notable finding was the positive trend between the magnitude of ADCC, antibody avidity, NAbs, and HAI responses observed at both pre- and post-challenge infection in vaccinates (**Table 1**). Mannose-conjugated inactivated whole SwIAV monovalent nanovaccine administered with or without a STING adjuvant generated high levels of cross-reactive neutralizing, HAI-, ADCC-, and CDC-mediating antibodies. Our data indicated that the level of HA gene identity between vaccine and challenge SwIAV potentially affects the breadth of antibody responses, and SwIAV vaccination using a nanovaccine candidate can induce both mucosal and systemic NAbs and nNAbs capable of clearing the heterologous SwIAVs from the respiratory tract.

## Data Availability

The original contributions presented in the study are included in the article/**Supplementary Material**. Further inquiries can be directed to the corresponding author.
