# Supplementary material for: Influenza-specific antibody-mediated and complement-dependent cellular cytotoxicity-inducing antibodies in vaccinated and infected pigs
Source: Front Immunol. 2025 Jun 30;16:1600761. doi: 10.3389/fimmu.2025.1600761 (PMC12256494; doi:10.3389/fimmu.2025.1600761)
Supplement: Supplementary file 1 [file DataSheet1.pdf]

Supplementary Table 1

	**Exp#1**	**Exp#2**
**Vaccine type**	Experimental Vaccine<br>Monovalent inactivated/killed whole SwIAV nanoparticle (chitosan or mannose-chitosan) based vaccine without adjuvant	Experimental Vaccine<br>Monovalent inactivated/killed whole SwIAV mannose-chitosan nanoparticle-based vaccine with adjuvant
	Commercial Vaccine (FluSure XP^{TM}, Zoetis, USA)<br>Multivalent Inactivated/killed whole SwIAV vaccine with adjuvant	Commercial Vaccine (FluSure XP^{TM}, Zoetis, USA)<br>Multivalent Inactivated/killed whole SwIAV vaccine with adjuvant
**Formulation**	Experimental Vaccine<br>▪ Chitosan-based NPs encapsulating whole inactivated/killed antigen (A/Swine/OH/FAH10-1/10 H1N2) (CS NPs-KAg)<br>▪ Mannose chitosan-based NPs encapsulating whole inactivated/killed antigen (A/Swine/OH/FAH10-1/10 H1N2) (mCS NPs-KAg)	Experimental Vaccine<br>▪ Mannose-chitosan NPs encapsulated with whole inactivated/killed virus (A/Swine/OH/FAH10-1/10 H1N2) plus ADU-S100 (STING adjuvant) (mChit-SwIAV + S100-eNP)<br>▪ Mannose-chitosan NPs surface absorbed with whole inactivated/killed virus (A/Swine/OH/FAH10-1/10 H1N2) plus ADU-S100 (STING adjuvant) (mChit-SwIAV + S100-sNP)
	Commercial Vaccine (FluSure XP^{TM}, Zoetis, USA)<br>Multivalent whole inactivated/killed antigen [SwIAV H1N1 (2 strains) + H1N2 (1 strain) + H3N2 (1 strain)] plus adjuvant (Amphigen)	Commercial Vaccine (FluSure XP^{TM}, Zoetis, USA)<br>Multivalent whole inactivated/killed antigen [SwIAV H1N1 (2 strains) + H1N2 (1 strain) + H3N2 (1 strain)] plus adjuvant (Amphigen)
**Dosage**	10^7 $TCID_{50}$ per dose	A/Swine/OH/FAH10-1/10 H1N2 antigen @ 250 µg (~10,000 HA units) plus ADU-S100 adjuvant @ 150 µg per dose
**Administration route**	Experimental vaccine – Intranasal mist	Experimental vaccine – Intranasal mist
	Commercial vaccine – Intramuscular	Commercial vaccine – Intramuscular
**Number of pigs and grouping**	Number of pigs total 19 (maternal antibody positive, n = 3 - 4 pigs per group)<br>Groupings:<br>Group 1 (n=3) - Mock (no vaccination and no challenge)<br>Group 2 (n =4) - Mock challenge (no vaccination and challenge)<br>Group 3 (n=4) - Commercial vaccine (FluSure XPTM, Zoetis) + challenge<br>Group (n=4) - CS NPs-KAg (10^7 $TCID_{50}$ equivalent of KAg from H1N2-OH10 virus per piglet + challenge<br>Group 5 (n=4) - mCS NPs-KAg (10^7 $TCID_{50}$ equivalent of KAg from H1N2-OH10 virus per piglet + challenge	Number of pigs total 30 (SPF, n =6 pigs per group)<br>Groupings:<br>Group 1 - Mock (no vaccination and no challenge)<br>(Group 2 - Mock challenge (no vaccination and challenge)<br>Group 3 - mChit-SwIAV + S100-eNPs + challenge<br>Group 4 - mChit-SwIAV + S100-sNPs + challenge<br>Group 5 - Commercial vaccine (FluSure XP^{TM}, Zoetis) + challenge

**Prime-boost intervals**	3 weeks	3 weeks
**Challenge**	Two weeks post booster vaccination	Two weeks post booster vaccination
**Challenge virus used**	Influenza A virus A/Swine/OH/24366/2007 H1N1 (H1N1-OH7)	Influenza A virus, 2009 pandemic CA09-H1N1 (A/California/04/2009)
**Challenge route**	Intranasal and intratracheal	Intranasal and intratracheal
**Sample collection time points**	Pre and post prime and booster vaccination at 0, 4 and 6 days post challenge	Pre and post prime and booster vaccination at 2, 4 and 6 days post challenge

Supplementary Table 2

**Candidate**	**Genotype (strain/lineage)**	**Lineage**	**HA gene identity (%) with challenge versus vaccine virus strain**
Experimental Vaccine (Exp #1 & Exp #2)	H1N2-OH10 (δ-lineage) (A/Swine/Ohio/FAH10-1/2010) [54]	δ-lineage	--
Commercial vaccine (Exp #1 & Exp #2)	H1N1 (02), H1N2 (01) and H3N2 (01)	--	93
Challenge virus (Exp #1)	H1N1-OH7 (A/Swine/OH/24366/2007) [55]	γ-lineage	77
Challenge virus (Exp #2)	CA09-H1N1 (A/California/04/2009) [56]	γ-lineage	78
